# Putting the pea in photoPEAriod

**DOI:** 10.1093/jxb/erac170

**Published:** 2022-06-24

**Authors:** Mark A Chapman

**Affiliations:** Biological Sciences, University of Southampton, Southampton SO17 1BJ, UK

**Keywords:** Domestication, flowering time, peas, photoperiod, quantitative trait loci

## Abstract

This article comments on:

**Williams O, Vander Schoor JK, Butler JB, Ridge S, Sussmilch FC, Hecht VFG, Weller JL.** 2022. The genetic architecture of flowering time changes in pea from wild to crop. Journal of Experimental Botany **73,**3978–3990.

**With an increasing human population, we are facing the need to grow more food, potentially expanding the environmental tolerances of our staple crops (** Godfray *et al.*, 2010; Campbell *et al.*, 2016**). The barriers to this include temperature, rainfall, soil type, daylength, and seasonality. In this issue, Williams *et al.*, in their study entitled ‘The genetic architecture of flowering time changes in pea from wild to crop’, advance our understanding of crop adaptation to photoperiod by revealing the genetic basis of photoperiod sensitivity in peas.**

Plants are adapted to their local environment in terms of the environmental cues (temperature and daylength) that promote or repress flowering, ensuring that this key physiological transition occurs at a time when the conditions are best suited for producing flowers, fruits, and seeds. Maladaptation (e.g. translocating a temperate crop to a tropical environment) would result in no flowering, or poorly timed flowering, and a reduction in yield. Based on the environmental cues plants use to transition into flowering, they are typically separated into long-day (LD), short-day (SD), and day-neutral types.

Understanding the genetic basis of flowering time, especially in relation to different environmental conditions, is a vital goal if we wish to expand the environments in which we can grow our crops (Cockram *et al.*, 2007; McClung, 2021). Mapping studies have indicated the quantitative genetic basis of this trait in many crops and, in a handful, we know the genes underlying the trait. Some patterns have emerged in the literature; for example, photoperiod evolution in different crops can be controlled by orthologous genes (e.g. maize *ZmCCT* and its rice orthologue *Ghd7*, and *PHYA* in common bean and soybean). Further, several photoperiod-related genes act in pathways that include *florigen/FT* genes, for example soybean *E1* and barley *ELF3*. In others, mutations affecting *FT* directly confer adaptive photoperiod responses and flowering induction under specific conditions (Box 1).

Box 1. ***FT* and its role in domestication across multiple crops**

The study by Williams *et al*. highlights paralogues of *FLOWERING LOCUS T (FT*) with roles in the modulation of the photoperiod response during the domestication of pea. In these, expression differences apparently underlie the different responses under the relevant conditions. *FT* is a well-known modulator of photoperiod sensitivity and response, acting downstream of circadian clock-regulated genes, and its expression promotes the expression of meristem identity genes in the shoot meristem. Whilst the pathway involves dozens of genes and proteins, interacting to control the initiation of flowering, *FT* (and its up- and downstream genes) is involved in the evolution of photoperiod response in many crops, thus permitting their expansion in other latitudes, which has been key to the expansion of agriculture from the sites of domestication (Cockram *et al.*, 2007; McClung, 2021). Some further examples include the following.

- In spring barley, a missense mutation in *Ppd-H1* delays flowering by preventing induction of *HvFT1* and reduces photoperiod responsiveness (Turner *et al.*, 2005).
- In tomato, a deletion in the enhancer region of *SP5G* (an *FT* orthologue) reduces expression and essentially removes photoperiod sensitivity (Soyk *et al.*, 2017).
- Two major haplotypes of SbFT12 in sorghum are differentiated by several sequence differences and these are distributed geographically with one common in tropical Africa (where plants typically exhibit an SD phenotype) and the other common in South Africa (where a day-neutral phenotype is most common). A paralogue (SbFT6) was also associated with photoperiod response (Cuevas *et al.*, 2016).
- Soybean has four *FT*-like paralogues, and *FT2c* shows a mutation specific to the domesticated lineage and present at very high frequency. This novel allele shows delayed flowering under SDs (Wu *et al.*, 2017).
- In sunflower, four *FT* paralogues have been identified, and only one, *HaFT1*, is expressed in the shoot apex. Wild plants flower earlier under SDs, and domesticated plants flower earlier under LDs. *HaFT1* in the domesticate has a frameshift (Blackman *et al.*, 2010).
- In maize, mutations in *ZCN8*, an *FT* orthologue, associate with the expansion further North from the origin of domestication in Central America and a parallel reduction in SD response (Guo *et al.*, 2018).
- In cucumber, large deletions upstream of *FT* (independently in Xishuangbanna-type and Eurasian populations) remove repression of FT expression and convert the crop to an earlier flowering phenotype. These deletions correspond to expansion into higher latitudes (Wang *et al.*, 2020).

Less well studied is the genetic basis of flowering time under SD and LD conditions in the same germplasm, and Williams *et al*. buck this trend, doing exactly this for field pea (*Pisum sativum*). Because some photoperiod-related genes are induced only under certain daylength environments, mapping quantitative trait loci (QTLs) for flowering in one environment probably identifies only a subset of the adaptive variation and does not allow for photoperiod sensitivity to be examined. Another advantage of the approach taken by Williams *et al*. is generating and examining a mapping population derived from crossing the wild progenitor (*P. sativum* ssp. *humile*, an LD plant incapable of flowering under SDs) to a cultivated accession (*P. sativum* ssp. *sativum*), thus identifying more about the domestication pathway and not simply the genetic basis of flowering in the domesticate. Variation found in wild progenitors (potentially bred out of cultivated accessions) may well be adaptive in a changing climate (McCouch *et al.*, 2013).

Pea is an important crop worldwide—approximately 9.7 M ha were grown in 2020 (FAO, 2020)—and a valuable model for studying adaptation to photoperiod which has clearly occurred post-domestication, allowing the latitudinal expansion of pea cultivation. Pea was one of the earliest plants domesticated in the Neolithic (Lev-Yadun *et al.*, 2000). In addition, the extensive study of inheritance patterns in peas by Mendel has led to several important genes being cloned (reviewed in Reid and Ross, 2011).

In the study by Williams *et al*., the recombinant inbred line (RIL) mapping population was grown under LD (16 h light) and SD (8 h light) conditions. The population was genotyped with >4500 markers, providing a good degree of coverage and short intermarker distances which aids in identifying candidate genes underlying QTL peaks. The genetic basis of photoperiod sensitivity has been examined in other germplasm, and three genes have been cloned that underlie this trait.

The first major finding was that five QTLs were identified with significant effects on days to flower (DTF) and were named *DTF1*, *3*, *5a*, *5b*, and *6* based on the chromosome they reside on (with two on chromosome 5). Three of these (*DTF1*, *5a*, and *6*) were previously identified in pea. Two of these QTLs (*DTF5a*, and *6*) have been cloned and the causative gene identified.

Some of the QTLs were followed up to identify candidate genes underlying each QTL. Firstly, *DTF5b* was shown to map close to the *LE* locus initially identified by Mendel, and, because of the similar phenotype observed (shorter stature, fewer nodes), it was assumed that DTF5b was equivalent to LE, a gibberellin 3β-hydroxylase (Lester *et al.*, 1997). *DTF1* and *3* were mapped in further populations wherein the target QTL was segregating but all other *DTF* QTLs were fixed for the wild allele; hence the role of the single QTL could be observed.

*DTF1*, although previously identified, had not been characterized at the molecular level. Williams *et al*. demonstrated that the domesticated allele in a wild background induced a domesticate-like phenotype (early flowering), and the wild allele induced a wild-like phenotype (late flowering) under both SD and LD conditions. Similar to the loci mapped above, after fixing other QTLs in the population, the marker at the peak of the QTL co-segregated with an *FT* paralogue, *FTa3*. Expression analysis of this gene revealed higher expression in plants with the domesticated allele than with the wild allele in both LD and SD conditions.

For *DTF3*, similarly the domesticated allele induced early flowering, but plants with this allele flowered at an intermediate time to the parental types. At the genomic location of the QTL peak was a cluster of *FT* genes. Again, examining expression, the authors were able to narrow down to one of these, *FT1a*, probably being causative because of its differential expression between plants carrying the domesticated and wild alleles.

The action of *TFL1c* which underlies *DTF6* was also followed up. Again, differential expression appeared to be causative in regulating the flowering response.

Whilst a mixture of expression and coding sequence changes are evident under previously identified domestication genes (Meyer and Purugganan, 2013), the genetic basis of domestication for altered flowering time in pea appears largely based on expression divergence. In the study of Williams *et al*., expression divergence between wild and domesticated pea was evident for the three genes examined which underlie *DTF1* (*FTa3*), *DTF3* (*FTa1*), and *DTF6* (*TFL1c*). The *FT* gene family is of clear importance in pea as well as in many other and diverse crops (Box 1). Mutations affecting gene expression are clearly more common than those affecting the coding region in the evolution of photoperiod response here and are apparent in many, but not all, other studies (Box 1).

A final and exciting observation is made by Williams *et al*. regarding a second, independent domestication of peas, recently characterized by Trněný *et al.* (2018). This domesticated subspecies, *Pisum sativum* ssp. *abyssinicum*, known as the Abyssinian pea or *dekoko*, is restricted to low latitudes in Ethiopia and Yemen, where days do not exceed 12.5 h in length and the ability to flower under SDs is therefore essential (Weller *et al.*, 2012). This ability must represent either an ancient expansion of a now-extinct lineage domesticated at higher latitudes, or an *in situ* domestication from a now-extinct wild population already adapted to lower latitude. In either case, it opens the door in peas for investigations into the parallel evolution of adaptation to novel photoperiods and other agronomically favourable phenotypes. Such studies would provide a complement to those in common bean which was also domesticated twice (Bitocchi *et al.*, 2013), and improve our understanding of parallel evolution under domestication in legumes more generally.
